# Evidence for spatially-responsive neurons in the rostral thalamus

**DOI:** 10.3389/fnbeh.2015.00256

**Published:** 2015-10-13

**Authors:** Maciej M. Jankowski, Johannes Passecker, Md Nurul Islam, Seralynne Vann, Jonathan T. Erichsen, John P. Aggleton, Shane M. O’Mara

**Affiliations:** ^1^Institute of Neuroscience, Trinity College DublinDublin, Ireland; ^2^School of Psychology, Cardiff UniversityCardiff, UK; ^3^School of Optometry and Vision Sciences, Cardiff UniversityCardiff, UK

**Keywords:** border cells, place cells, head direction cells, rostral thalamus, parataenial nucleus, anteromedial nucleus, nucleus reuniens

## Abstract

Damage involving the anterior thalamic and adjacent rostral thalamic nuclei may result in a severe anterograde amnesia, similar to the amnesia resulting from damage to the hippocampal formation. Little is known, however, about the information represented in these nuclei. To redress this deficit, we recorded units in three rostral thalamic nuclei in freely-moving rats [the parataenial nucleus (PT), the anteromedial nucleus (AM) and nucleus reuniens NRe]. We found units in these nuclei possessing previously unsuspected spatial properties. The various cell types show clear similarities to place cells, head direction cells, and perimeter/border cells described in hippocampal and parahippocampal regions. Based on their connectivity, it had been predicted that the anterior thalamic nuclei process information with high spatial and temporal resolution while the midline nuclei have more diffuse roles in attention and arousal. Our current findings strongly support the first prediction but directly challenge or substantially moderate the second prediction. The rostral thalamic spatial cells described here may reflect direct hippocampal/parahippocampal inputs, a striking finding of itself, given the relative lack of place cells in other sites receiving direct hippocampal formation inputs. Alternatively, they may provide elemental thalamic spatial inputs to assist hippocampal spatial computations. Finally, they could represent a parallel spatial system in the brain.

## Introduction

The anterior thalamic nuclei appear vital for human episodic memory (Aggleton and Brown, 1999; Harding et al., 2000; Carlesimo et al., 2011), while these same thalamic nuclei in rodents are required for spatial learning (Byatt and Dalrymple-Alford, 1996; Aggleton and Nelson, 2015). Disconnection evidence shows that the anterior thalamic nuclei function in close concert with the hippocampus (Parker and Gaffan, 1997; Warburton et al., 2001; Henry et al., 2004). There is, however, a longstanding need to determine the class of information processing performed by the anterior and adjacent rostral thalamic nuclei, and why it is necessary for structures such as the hippocampus (Sutherland and Rodriguez, 1989; Aggleton et al., 2010). To address these questions, the present study examined the electrophysiological properties of rostral thalamic cells in awake, behaving rats. Rostral thalamic cells were found with spatial properties that appear to mirror those found in hippocampal and parahippocampal regions, thereby offering new perspectives into the importance of this diencephalic region for spatial learning and memory.

The anterodorsal thalamic nucleus provides hippocampal head direction information (Taube, 1995, 2007; Goodridge and Taube, 1997; Tsanov et al., 2011b). This function is not, however, sufficient to explain the importance of the anterior thalamic nuclei for spatial processes, as shown by the mild effects of lateral mammillary nuclei lesions (Vann, 2005, 2011), which disconnect head direction information from the anterior thalamic nuclei (Sharp and Koester, 2008). While the anteroventral thalamic nucleus is a potential source of hippocampal theta (Vertes et al., 2001; Albo et al., 2003), this additional role is again unlikely to explain the full impact of anterior thalamic lesions as other sites also provide hippocampal theta (Woodnorth et al., 2003; Vertes et al., 2004). Furthermore, lesions of the anterior thalamic nuclei that spare the anteromedial nucleus produce only partial deficits on tests of spatial learning (Aggleton et al., 1996; Byatt and Dalrymple-Alford, 1996; Van Groen et al., 2002; Peckford et al., 2014), suggesting additional contributions from the AM (Aggleton et al., 2010).

To examine these potential contributions, adult rats were implanted with tetrodes directed at the AM. Further recordings were made in two adjacent sites, the parataenial nucleus (PT) and nucleus reuniens (NRe). The PT is poorly understood, though it is known to have dense frontal connections, as well as inputs from the ventral subiculum (Van der Werf et al., 2002). NRe has been implicated in the persistence of spatial memory (Dolleman-van der Weel et al., 2009; Hembrook and Mair, 2011; Loureiro et al., 2012), presumably reflecting its role as a relay for prefrontal-hippocampal connections (Herkenham, 1978; Vertes et al., 2006; Prasad and Chudasama, 2013). NRe also possesses a population of head direction cells, which we have described elsewhere (Jankowski et al., 2014). The finding that the addition of NRe damage to an anterior thalamic lesion exacerbates the resulting spatial deficits (Warburton et al., 1999) supports the idea that the anterior thalamic nuclei and NRe have related, but different, spatial properties. These properties remain, however, to be defined.

## Materials and Methods

### Animals

Nine (of twelve; 4–6 months) male Lister-Hooded rats (Bantin and Kingman, UK) weighing 420–530 g provided the data to be described. Additional recording data from three other rats were consistent with the present findings but are not described because their electrode tracks were more difficult to reconstruct. Their exclusion reflects several difficulties: there was greater tissue distortion in the septal hippocampus and rostral thalamus, reflecting the technical challenges of executing such deep recordings, and because aspects of the electrode tracks were not visible, despite the known excursion of the electrodes themselves, so impacting on the anatomical reconstructions (see “*Histological Analyses*” Section).

### Ethics

Upon arrival in the laboratory, animals were housed individually and handled by the experimenter daily for a week before being trained in the pellet-chasing task (see below). Rats were food-restricted to 85% of their *ad libitum* body weight and kept in a temperature-controlled laminar airflow unit and maintained on a 12 h light/dark cycle (lights on from 08 to 20 h). Experiments were conducted in accordance with European Community directive, 86/609/EC, and the Cruelty to Animals Act, 1876, and were approved by the Comparative Medicine/Bioresources Ethics Committee, Trinity College, Dublin, Ireland, and followed LAST Ireland and international guidelines of good practice. Surgery was conducted under isoflurane anesthesia, an appropriate post-surgery monitoring and analgesia regime was in place, and every effort was made to minimize suffering.

### *In vivo* Electrophysiology and Surgery

**Figure 1:**
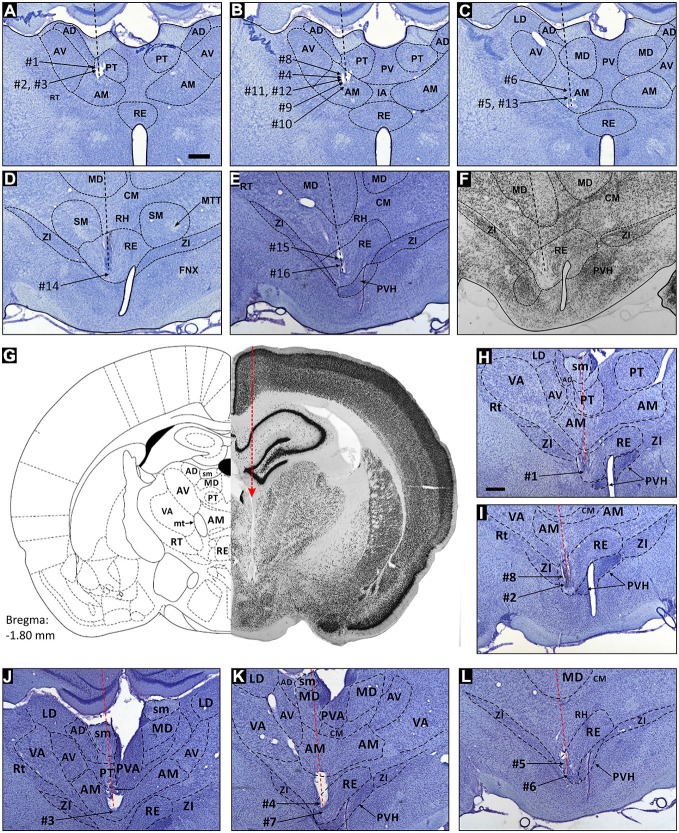
**Reconstruction of electrode tracks and place cells in the parataenial, anteromedial and reuniens thalamic nuclei**. Location of spatial cells in the rostral thalamic nuclei reconstructed on coronal sections (Nissl stain). Units from the parataenial nucleus (PT) of the thalamus are cells 1–3, and their reconstructed locations on histological slide are depicted on **(A)**. Units from anteromedial nucleus (AM) of the thalamus are cells 4–13, and their locations are reconstructed on **(B,C)**. Units from nucleus reuniens (NRe) are cells 14–16, and their estimated locations are presented on **(D,E)**. **(F)** NeuN stained brain slice, which is the adjacent section to slice presented in **(E)** and comes from the same animal. **(G)** Presents a hemi-coronal reference section (left-hand side), and the corresponding hemi-coronal histological section. **(H)** and **(I)** are enlarged slices stained for Nissl substance with reconstructed recording position of perimeter/border cells 1, 2 and 8 in NRe of the thalamus. Histology in **(G)** comes from the same animal as presented in **(H)**. **(J)** Location of perimeter/border cell 3, which was recorded in the ventral part of anteromedial nucleus of the thalamus. **(K,L)** Location of perimeter/border cells 4–7 recorded in NRe of the thalamus. Scale bar is 500 μm. Abbreviations: AD, anterodorsal thalamic nucleus; AM, anteromedial thalamic nucleus; AV, anteroventral thalamic nucleus; CM, central medial nucleus; FNX, fornix; IA, interanteromedial thalamic nucleus; LD, laterodorsal nucleus; MD, mediodorsal thalamic nucleus; MTT, mammillothalamic tract; PT, parataenial nucleus; PV, paraventricular thalamic nucleus; PVH, paraventricular hypothalamic nucleus; RE, nucleus reuniens; RH, rhomboid nucleus; RT, reticular nucleus; SMT, submedial thalamic nucleus; VA, ventral anterior thalamic nucleus; ZI, zona incerta.

Descriptions of the surgical protocol and recording techniques can be found elsewhere (e.g., Brotons-Mas et al., 2010; Tsanov et al., 2011b). Briefly, rats were implanted with a bundle of eight tetrodes of ø 25 μm platinum–iridium wires (California Fine Wire Ltd., CA, USA) mounted onto small drivable 32-channel microdrives (Axona Ltd., UK) targeted at the rostral thalamus (specifically, the PT, the AM and NRe; see Figure 1 for sample histological verification of the recording sites). The coordinates were as follows: 1.60 mm posterior to bregma, 1.20 mm lateral to the midline and at an angle of about 5.5°. Depth varied depending on the target structure and ranged from 4.8–5.6 mm below the brain surface (Figure 1). The anatomical plane chosen for electrode incursion was selected so that electrodes might pass through several nuclei of interest to reach NRe, including the PT prior to reaching AM. The small mediolateral extent of these nuclei (~from approximately 0.5–1.5 mm; see scale bar, Figure 1) highlights the difficulty of precisely targeting these sites. The coordinates were also chosen to enter the rostral thalamus through the stria medullaris thalami and so pass on the medial side of the AD thalamic nucleus to avoid possibility of contamination of our data set with HD cells from this thalamic nucleus. Rats were allowed at least 1 week of recovery post-surgery. Tetrodes were lowered slowly through the brain (maximal rates 25–50 μm/day), typically over a period of weeks to prevent tissue damage and to ensure successful rostral thalamic electrode targeting and penetration. Recordings from these nuclei present special challenges because of their small size and mid-line placement. Based on the daily record of the electrode position and post-mortem histological verification, each recording could be located along the tetrode track, with respect to the planned direction of incursion, as determined from the atlas. The recording sessions took place in partially-curtained arenas located in the center of the test room, which contained multiple, large visual cues made salient to allow the animals to orient themselves in the environment.

### Apparatus

Experiments were conducted in a circular arena (diameter 96 cm). The insides of the arenas were uniform matt black. Low-level lighting was used during light testing. The environment was partially curtained, with a visual cue card (an A4 sheet with a geometric pattern) in a constant location on the inside of the curtain. All experiments were conducted during the day between 09 and 20 h. Session lengths were typically 20 min. Rats performed a pellet-chasing task during the course of the experiments. During testing, 20 mg food pellets (TestDiet^TM^, 5TUL formula, TestDiet, USA) were thrown in the arena at random locations approximately every 20 s. During the weeks of recordings, animals were allowed 20 g of food daily.

### Histological Analyses

On completion of the recording studies, the rats received an overdose of anesthetic [1.5 g of urethane (Sigma-Aldrich) dissolved in 4.5 ml water] and were then perfused intracardially with 250 ml of 0.1 M phosphate-buffered saline (PBS) at room temperature followed by 350 ml of 4% paraformaldehyde in 0.1 M PBS at ~4°C. The brains were then removed and placed in 4% paraformaldehyde (for at least 72 h). Brains were blocked, placed on a freezing platform, and 40 μm coronal sections were cut with a sledge microtome (Leica 1400). Two alternate series that used all sections were taken through the rostral thalamus. One series was mounted directly onto gelatine-subbed slides, allowed to dry overnight, then stained with cresyl violet, a Nissl stain. The second series was immunologically-reacted with the neuronal marker α-NeuN (MAB 377, Chemicon, Watford, UK), then with a secondary horse anti-mouse rat adsorbed antibody (AI-2001, Vector Laboratories Ltd., Peterborough, UK) and subsequently visualised with Vector Elite ABC (PK-6100, Vector Laboratories Ltd.) and diaminobenzidine. The NeuN stain makes it possible to visualise neurons in the absence of glial cells (Jongên-Relo and Feldon, 2002), so helping to identify cytoarchitectonic features. A Leica DM5000B microscope with Leica DFC310FX digital camera and Leica Application Suite image acquisition software was used for brightfield microscopy.

Recording positions were determined by calculating the distance above the deepest electrode position and calculating the distance below the first penetration into the tissue. Given the depths of the nuclei we are attempting to record from, we occasionally found that the electrodes sometimes caused a small degree of tissue distortion (Figure 1), which was compensated for in the position calculations. Positions of recorded cells were estimated as follows: theoretical positions of electrodes tips and rostral thalamic borderlines were estimated by reference to Swanson (1992) and the reconstructed histological specimens; tissue distortion was adjusted for when making the position calculations. The degree of distortion could be estimated by working from the size and positon of the nuclei in the standardized atlas and the coronal sections we prepared. We required that the nuclei had to remain intact, that there was no substantial distortion of nuclei as determined by visual estimates from the atlas or comparable histological sections and finally, that there was little or no exfiltration of blood from any blood vessels. The position of the electrodes below the brain surface was determined for each recording session and expressed in μm, thereby allowing estimates of each cell position to be derived. In essence, the reconstructions proceed from a standardised estimation of nuclear positions from the histological atlases, the known lengths and excursions of the electrodes, and working between the histological slides and the atlas to ensure we obtain as accurate a reconstruction as it is possible to provide. We note here that, we have conducted many histological reconstructions along with the relevant electrode tracks, and we are providing what is our best estimate of the terminal position and trajectory of the electrode. The electrode is implanted at an angle relative to the coronal position of the brain, and therefore it is not possible to cut coronal sections that are parallel to the electrode trajectory; rather, the coronal sections will intersect with the trajectory of the electrode in the brain. Thus, the position of the electrode track through the brain and the coronal sections appear at a relative angle to each other. The coronal sections do allow us to be precise about nuclear boundaries despite the small, relative distortions caused by the electrode track.

### Recording and Statistical Analysis

Standard statistical testing using a custom-written suite of Matlab scripts (NeuroChaT^TM^) and Axona software. Unit identification involved several criteria. First, units had to be active and had to show consistent waveform characteristics (amplitude, height, and duration) during the recording session. The amplitude of each spike was measured as the difference between the positive peak and first negative peak before the positive peak, if present, or zero. The height was measured as the difference between the peak to the minimum value of the spike waveform. The width of the spikes was determined as the distance in microseconds beyond which the waveform drops below 25% of its peak value. Furthermore, units had to demonstrate a clean refractory period (>2 ms) in the inter-spike interval (ISI) histogram. Units were sorted using conventional cluster-cutting techniques (Tint, Axona Ltd.). Units were classified based on the spatio-temporal features of their activity in the open field environment where they were recorded during the pellet chasing task. Units were sometimes seemingly recorded for more than for 1 day, despite lowering of the electrodes. In those cases, during the final spike sorting, cells were monitored on the relevant tetrodes from day to day; for analysis, only clean recordings with the biggest sample size and spikes of the highest amplitude were chosen, and particular care was taken to exclude seemingly-related samples from analysis to avoid inadvertent double-counting of cells. During spike sorting, the signals from each cell were carefully followed from first appearance to complete loss, in order to avoid overestimation in the cell counts. Additionally, any ambiguous sessions were rejected. Once well-defined neuronal signals were isolated recording commenced. Additionally, rats had to explore at least 90% of the open field in a session to be included in analyses to allow reliable calculation of spatial characteristics).

### Spatial Analyses

Additional analyses examined spatial modulation of recorded units. We used multiple indices to analyze the spatial properties of the hippocampus place cell firing (namely spatial coherence, spatial information content, and spatial reliability). A firing field was defined as a set of at least nine contiguous pixels with firing rate above zero. A place field was identified if nine neighboring pixels (sharing a side) were above 20% of the peak firing rate. Place field size was represented by number of pixels. The spatial specificity (or spatial information content) is expressed in bits per spike (Skaggs et al., 1996). The spatial selectivity of a firing field (ratio of maximal signal to noise) was calculated by dividing the firing rate of the cell in the bin with the maximum average rate by its mean firing rate over the entire apparatus (Skaggs et al., 1996). Mean frequency is the total number of spikes divided by the total recording time and is expressed in Hz. Exploration was assessed by comparing the occupancy of bins and the number of visits per bin during recording sessions. Additionally, to be regarded as place cells, the following criteria had to be met: all included as place cells had to have a spatial information content (Skaggs et al., 1996) index of > 0.5; a spatial coherence of > 0.25; and a mean firing rate > 0.25.

### Head Direction (HD) Analyses

Directional analyses were performed for all recorded cells in the AM (36 head direction units recorded in total). Head direction cells observed in NRe are the subject of a separate publication, and these data are not reported here (Jankowski et al., 2014). The rat’s HD was calculated for each tracker sample from the projection of the relative position of the LEDs onto the horizontal plane. The directional tuning function for each cell was obtained by plotting the firing rate as a function of the rat’s directional heading, divided into bins of 5°. The firing rate was computed based on the total number of spikes divided by the total time in that bin (Taube, 1995). To restrict the influence of inhomogeneous sampling on directional tuning, data were accepted only if all directional bins were sampled by the rat. The directionality of the HD units in the horizontal plane (measured in degrees) was normalized for comparison of the HD firing rate properties. The peak firing rate of cells that respond to a different direction of heading was aligned to a HD of 180°. The firing rate was normalized (with values between 0 and 1) with respect to the peak firing rate for each unit. The firing rate is calculated by dividing the number of spikes by the number of visits at a particular head direction bin. The clockwise/counter-clockwise (CW/CCW) separation was calculated by considering a particular angular head velocity threshold (120°/s). Thus, when the rat moves at +120°/s, it was placed into the CW head direction category (similarly, for CCW, −120°/s).

### Perimeter/Border Cell Analyses

Perimeter or border cells were considered to be units that have a narrow curvilinear firing profile parallel to the circumference of the recording arena (a circular arena), creating a concentric circle (or arc) adjacent to the physical wall of the environment (We use the phrase “perimeter” to acknowledge the possibility that these units may not be responding to a border between components of an environment, but simply to the limit of the environment itself). We therefore applied a dual approach based on stated criteria to allow us to define a perimeter/border cell, as well as a new metric which allows us to quantify the degree to which a unit could be described as a perimeter/border cell. Our metric also allowed reliable discrimination between perimeter/border cells and place cells that happen to be adjacent the boundary of the arena and are distorted by the presence of the boundary (because there are some positions the rat is unable to physically occupy, giving a non-isotropic firing field).

Perimeter/border cells were first considered as cells with a visually-apparent firing profile parallel to the physical boundary (or circumference) of the recording arena, determined by visual inspection after plotting the positional data. Perimeter/border cells were then quantified using the following criteria: the firing field should be a continuous arc of 90° or greater, while at least 80% of the spiking activity had to present within the limits of two concentric circles. The outer circle or border is the physical boundary of the arena determined from the raw position data. The inner circle was determined as follows: pixels with at least 20% of the maximum firing rate were considered as active pixels. We then used taxicab geometry (city block distance) to compute the smallest taxicab distance of the active pixels from the outer circle (all calculations were made using MatLab). The peak distance (distance at which maximum number of active pixels are found) and the standard deviation of the distance were used to compute the truncated normal distribution along the radial distance from the outer circle (with a uniform circular distribution along the arc). Thus, all the pixels equidistant from the border have the same probability of being present. The inner circle was then determined as the largest distance from the outer circle towards the center for which the probability is greater than 0.01 [the probability that a pixel at (i, j) will fire with probability P(i, j) when it is at a distance d(i, j) from the border].

The inner and outer circles were used as the concentric circles for further analysis. The percentage of spiking activity was calculated from the number of active pixels within these circles divided by the total number of such active pixels over the entire arena. The angular distance of the active pixels within the circles was calculated from the center of the arena. A histogram of the angular distance was then prepared. The angular extent of the firing field is the largest segment of the histogram with a non-zero count of angular distance. This approach then makes it possible to eliminate place cells that happen to fire adjacent to the wall but have a curvilinear appearance because the physical constraints of the test arena create seemingly elongated fields. In this case, there will be a high probability of spiking which might coincidentally meet the criteria for (say <45°), but not for longer arcs (say >90°). This effect will be especially apparent on the rotational autocorrelations, where the probability shows sudden rises and falls for place cells, but not for perimeter/border cells meeting these criteria.

### Autocorrelation Histograms of the ISI Distribution

These were obtained for ISI lags of −1000 to +1000 ms between spikes with 1 ms-wide bins. The non-normalized autocorrelation signal was fitted to Equation (1) (an extended form of the one used by Brandon et al., 2013):

(1) $$y(x)=[a_{1}\cos(\omega_{1}x)+a_{2}\cos(\omega_{2}x)]\;\times \;\exp\left(-\frac{\left|x\right|}{\tau_{1}}\right)+b+c_{1}\exp\left(-\frac{\left|x\right|}{\tau_{2}}\right)-c_{2}\exp\left(-\frac{\left|x\right|}{\tau_{3}}\right)$$

Here, *y* is the autocorrelation signal, x is the lag, a_1_ and a_2_ are the amplitude of the oscillating terms with frequency ω_1_ and ω_2_ modulated by exponential with decay constant τ_1_.The terms τ_2_ and τ_3_ are the decay constants for the exponentials with amplitude c_1_ and c_2_. The parameters a_1_, a_2_, b, c_1_ and c_2_ were allowed to vary in the range [0, N], where N is the peak of the autocorrelation signal. The range of decay constants are, τ_1_ = [0, 5000], τ_2_ = [0, 100], τ_3_ = [0, 10] in millisecond unit. ω_1_ and ω_2_ were varied in the range [12π, 24π] and [6π, 12π], respectively.

Assuming spikes were generated following a Poisson process, the ISI was considered to follow an exponential distribution, which also gives an exponential distribution in its autocorrelation, represented in Equation (1) by the positive exponential component comprised of (c_1_, τ_2_). The initial dip in the autocorrelation for the refractory period in the ISI was given by the fast-decaying negative exponential (c_2_, τ_3_). The alternating low and high peaks are modeled as the superposition of two oscillations, given by two slow decaying cosine functions, as if the high peaks are generated when the oscillations are in the same-phase and the low peaks are generated when they are in anti-phase. The baseline shifts for the cosine terms and all the constant errors are interpreted in constant b. The curve fitting followed the measurement of “jump factor”, defined as the relative contribution of the low frequency components in the higher peaks given by a_2_/(a_1_ + a_2_). Frequency ratios of the cosine functions were also measured to verify the superposition model of the Equation (1).

#### Theta Skipping Cell Analyses

The theta cycle skipping index (TS) was measured using Equation (4) of Brandon et al. (2013), see also Deshmukh et al. (2010). As described (Brandon et al., 2013), the TS range should be [−1 to +1]. A positive TS is present if the second peak after the center peak is larger than the first one; a negative TS is not expected according to the model in Equation (1) if it is fitted for the theta-skipping cycle cell with alternative low and high peaks. We did not find any negative TSs. The greater the TS, the greater the larger peak is jumped from the theta-modulated signal, caused by the interference of the second oscillation. We measured this effect with an alternative index “jump factor” defined as a_2_/(a_1_ + a_2_), which provides a direct measurement of the relative contribution of the two oscillations. A jump factor >0.5 means the contribution of the slow varying interfering oscillation in the jump is greater than the theta-range oscillation. If the frequency ratio follows a 2:1 ratio (or closer), then the mathematical basis of using two cosine functions of different frequency ranges to obtain consecutive in-phase or anti-phase superposition to yield lower and higher peaks, respectively, is verified.

### Statistical Analyses

Statistical significance was estimated by using ANOVA and *post hoc* tests. The probability level interpreted as statistically significant was *p* < 0.05.

## Results

### Histology and Cell Numbers

In total, 939 well-isolated units were recorded in nine rats and assigned, after post-mortem histological verification as follows: NRe (*n* = 544 units), the AM thalamic nucleus (AM, *n* = 371), and the PT (*n* = 24). To select animals for analysis, we set the following criteria: histologically-verified electrode tracks should be confirmed as having traversed the stria medullaris and/or PT, the AM and/or the NRe. Electrode tracks were localized predominantly in the mid anterior-posterior portions of AM and PT, while typically traversing the more lateral and ventral parts of NRe. The position of recorded units is indicated by the numbers on the inset electrode track reconstructions on the histological specimens in Figure 1. We have marked our best estimates of nuclear boundaries on the representative histological specimen; similarly, we mark the final detectable termination point of the tetrode track with respect to the nuclear boundaries, using the methods described above. In none of these cases did we observe tissue from hippocampal (or parahippocampal) regions pulled into the deep thalamic regions from which we were recording. Inspection of the representative specimens presented in Figure 1 confirms that the inferior aspect of the hippocampal formation remains intact, thus we can exclude the presence of ectopic or explanted tissue as the explanation for the observed phenotypes of the units described here. In the specimen cases presented here, the electrode tracks were clearly visible, and could be recovered in histological section. In all cases, the electrode tracks visibly penetrate and cross the relevant nuclear boundaries in rostral thalamus, namely, the AM, the PT and NRe. Thus, our chosen implantation coordinates allowed us to access the relevant nuclei, despite their depth within the brain. We found that the lateral ventricle or the stria medullaris provided a useful anatomical point of reference, as there were no units present in this fraction of the electrode trajectory, although unit activity would have been present at more dorsal locations. The resumption of unit activity at more ventral locations provided the necessary indication that the electrodes were present in the targeted rostral thalamic nuclei.

**Figure 2:**
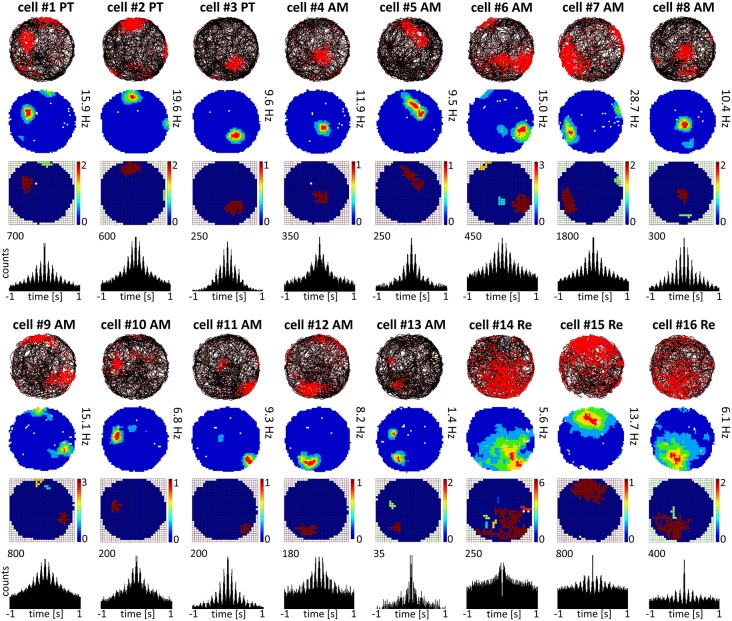
**Place cells recorded in parataenial, anteromedial and reuniens thalamic nuclei**. Place cells with consecutive numbers are presented in columns. For each unit, data recorded during the whole 20-min sessions are presented: the path of the animal with superimposed unit firing activity, firing intensity map, place field map and autocorrelation ±1000 ms depicting rhythmic firing in the 6–12 Hz range observed in the majority of recorded place cells.

The electrophysiological properties of the thalamic units with spatial properties are presented in Table 1 (single unit activity is provided on insets on Figures 2, 4, 6; more details are provided in Figure 8, see below). The numbers and percentages of cells recorded in PT (three rats) were: place cells 7 (29.2%); cells with activity modulated by 6–12 Hz oscillations 4 (16.6%); unidentified low-firing units 13 (54.2%). The numbers and percentages of cells recorded in AM (seven rats) were: place cells 23 (6.2%); head direction cells 36 (9.7%); perimeter/border cells 2 (0.5%); theta cycle skipping cells 4 (1.1%); cells with activity modulated by 6–12 Hz oscillations 32 (9.7%); fast-firing cells 12 (3.2%); unidentified low firing units 260 (70.1%). It should be noted that the electrode tracks repeatedly traversed AM but were not so medial as to involve the inter AM (e.g., Figures 1A–F). The numbers and percentages of cells recorded in NRe (six rats) were: place cells 11 (2.0%); perimeter/border cells 11 (2.0%); head direction cells were described separately (Jankowski et al., 2014); theta cycle skipping cells 19 (3.5%); cells with firing activity modulated by 6–12 Hz oscillations 79 (14.6%); fast-firing cells 24 (4.4%); unidentified low-firing units 340 (62.5%). The theta cycle skipping cells were characteristically found in NRe, where they were electrophysiologically co-localized with head direction cells that have been described separately (Jankowski et al., 2014), and that separate population of head direction cells is not reported here. A total of 613 cells (65.3%) across all rats were classified as unidentified low firing units—cells that did not appear to exhibit any particular temporal or spatial properties or formed groups smaller than four cells with similar phenotype.

**Table 1:** **Electrophysiological classification of thalamic units with spatial properties (mean ± SEM)**.

Region		Place cells	HD cells	Perimeter/border cells
Parataenial nucleus	N	7	NA	NA
	Mean spike amplitude	131.7 ± 21.7 μV	NA	NA
	Mean spike width	151.0 ± 28.1μs	NA	NA
	Mean hight	172.9 ± 56.2 μV	NA	NA
	Mean frequency	1.15 ± 0.25 Hz	NA	NA
	Maximal place map frequency	8.98 ± 2.46 Hz	NA	NA
	Spatial coherence	0.75 ± 0.07	NA	NA
	Spatial information content (Skaggs)	2.12 ± 0.47	NA	NA
Anteromedial nucleus	N	23	36	2
	Mean spike amplitude	165.9 ± 18.9 μV	101.8 ± 3.3 μV	NA
	Mean spike width	133.3 ± 7.6 μs	116.0 ± 1.7 μs	NA
	Mean hight	213.4 ± 29.0 μV	130.5 ± 6.8 μV	NA
	Mean frequency	1.90 ± 0.67 Hz	4.90 ± 1.01 Hz	NA
	Maximal place map frequency	11.41 ± 2.78 Hz	11.15 ± 2.34 Hz	NA
	Spatial coherence	0.70 ± 0.03	0.61 ± 0.02	NA
	Spatial information content (Skaggs)	2.17 ± 0.22	0.66 ± 0.07	NA
Nucleus reuniens	N	11	NA	11
	Mean spike amplitude	165.3 ± 28.2 μV	NA	133.6 ± 30.8 μV
	Mean spike width	109.2 ± 8.3 μs	NA	126.3 ± 5.6 μs
	Mean hight	178.5 ± 25.5 μV	NA	160.1 ± 27.7 μV
	Mean frequency	3.11 ± 1.03 Hz	NA	1.13 ± 0.39 Hz
	Maximal place map frequency	13.92 ± 6.3 1 Hz	NA	5.68 ± 1.50 Hz
	Spatial coherence	0.66 ± 0.07	NA	0.44 ± 0.03
	Spatial information content (Skaggs)	0.97 ± 0.13	Jankowski et al. (2014)	1.42 ± 0.30

*Summary statistics for mean spike amplitude, width and height, mean frequency, maximal place map frequency, spatial coherence and spatial information content for all spikes (mean ± SEM) for the three groups of units expressing spatial properties in the anteromedial (AM) and parataenial (PT) nuclei, and in nucleus reuniens (NRe). ANOVA followed by Tukey’s tests and multiple two-tailed t-test for paired two samples for means with Bonferroni correction indicates that place cells in NRe have a significantly lower spatial information content (*p* < 0.05) compared to place cells recorded from AM or PT. There were no differences in spatial information content between PT and AM (one-way ANOVA followed by Tukey’s). (Skaggs) refers to Skaggs et al. (1996). HD, head direction cells*.

### Place Cells are Present in the Parataenial, Anteromedial, and Nucleus Reuniens of the Thalamus, and are not Topographically-Organised

**Figure 3:**
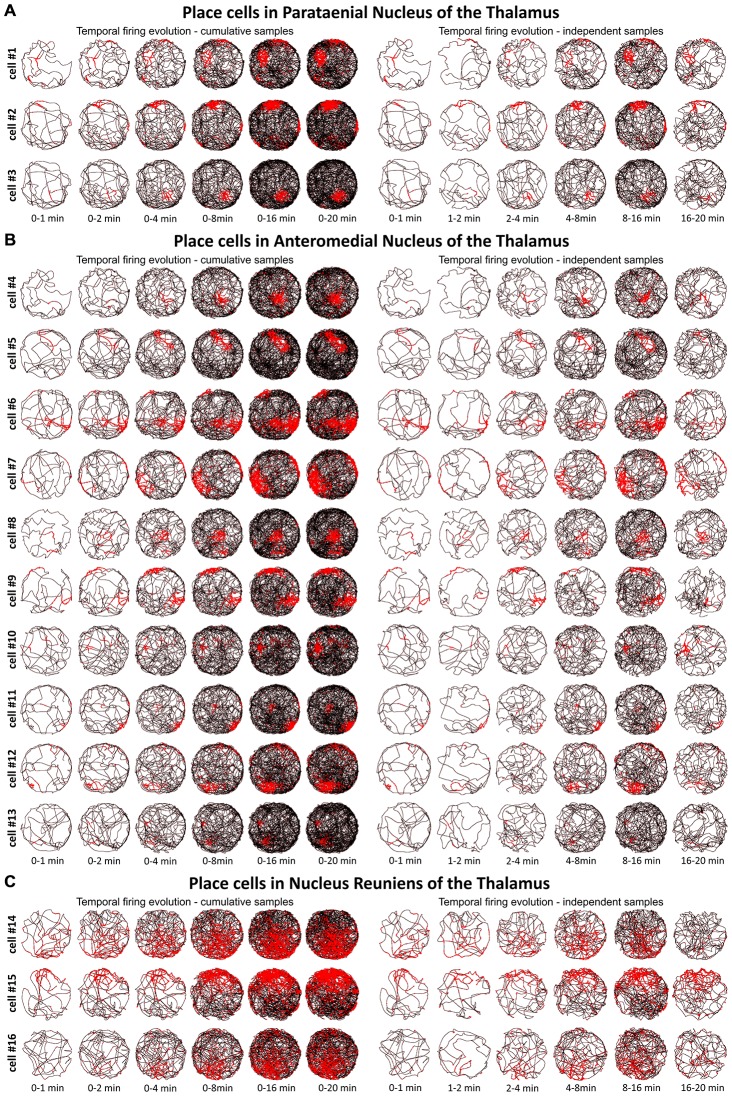
**Temporal evolution of the discharge profile of thalamic place cells. (A)** The temporal evolution of spatial firing in place cells recorded in the PT of the thalamus. Cells 1–3 are presented in rows for two columns of six time-binned samples for cumulative (on the left) and independent (on the right) time intervals. **(B)** The temporal evolution of spatial firing in place cells recorded in the AM of the thalamus. Cells 4–13 are in time-binned samples for cumulative (on the left) and independent (on the right) time intervals. **(C)** The temporal evolution of spatial firing in place cells recorded in the NRe of the thalamus. Cells 14–16 are presented as rows of six time-binned samples for cumulative (on the left) and independent (on the right) time intervals. All place cells on this figure correspond with cell numbers presented on Figure 2.

Cells exhibiting the characteristic phenotype that passes threshold for classical hippocampal place cells were found in PT, AM and NRe (examples of these cells are presented in Figure 2 and single-unit activity in Figure 8). Cells recorded in PT (see examples of cells #1–3 on Figure 2) and AM (#4–13) had relatively sharp and small place fields, compared to cells recorded in NRe (#14–16), as shown on Figure 2 on maps with raw spiking activity superimposed on the path of the animal, firing intensity maps and on place field maps. NRe cells were characterized by generally higher spontaneous activity outside the center of the place field and thus wider receptive fields. Place cells found in NRe had significantly lower spatial information content than those from PT or AM (*p* < 0.05) as tested in one way ANOVA followed by Student-Newman-Keuls *post hoc* test. Moreover, place cells recorded in PT and AM sometimes formed two or three place fields, unlike NRe cells which usually fired mainly at single somewhat less precisely-defined location. Activity of majority of cells from all three nuclei was entrained by 6–12 Hz oscillations as depicted on ±1000 ms autocorrelograms (Figure 2). However, the firing activity of some units in NRe did not show any rhythmicity (e.g., cell #14, Figure 2). All place cells show sharply-defined, location-specific firing that remains stable and constant across the duration of the recording session (Figure 3). The recorded place cells do not appear to exhibit a topographic organisation because simultaneously-recorded cells (e.g., on Figure 2 see pairs of cells 5 and 13 or 11 and 12 recorded simultaneously in AM or cells 2 and 3 recorded simultaneously in PT) all show place fields that are distal to each other within the recording arena.

### Place Cells Fire from First Exposure to an Environment

We tested time of onset of first firing for our place cell samples. A delayed onset might suggest (Hok et al., 2012) that there is either instability of place representation or an online remapping of place representation is occurring. Figure 3 depicts the temporal evolution of spatial firing in the PT, AM, and NRe for cumulative samples (left-hand columns) and for independent time-binned samples (right-hand columns). Spatial activity is present from the first minute of exposure to the environment, indicating that there is a rapid or near-instantaneous expression of place information by these thalamic cells.

### Perimeter/Border Cells are Present in Nucleus Reuniens of the Thalamus

**Figure 4:**
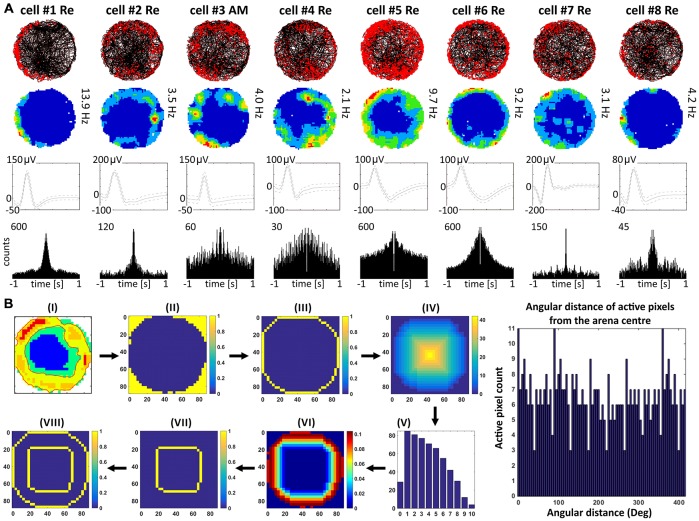
**Perimeter/border cells recorded in nucleus reuniens and anteromedial nucleus of the thalamus. (A)** Perimeter/border cells with consecutive numbers are presented in columns. For each cell, data recorded during the entire 20-min session are presented: the path of the animal with superimposed unit firing activity, firing intensity map, spike waveform, and autocorrelation ±1000 ms. **(B)** Analytical steps to test for the presence of border cells. (i-viii) Steps from visual identification of putative border cell, to automated fitting of concentric circles based on external border to classification of cell type (see Materials and Methods for a full description).

**Figure 5:**
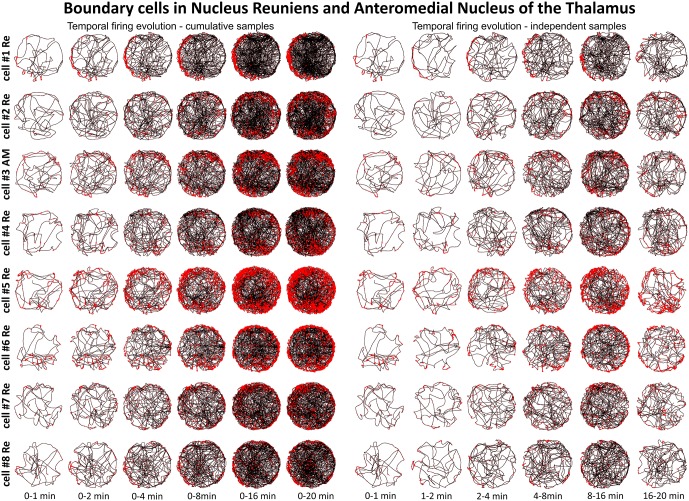
**Temporal evolution of the discharge profile of thalamic perimeter/border cells**. The temporal evolution of spatial firing in perimeter/border cells recorded in the NRe and AM of the thalamus. Cells 1, 2, 4, 5, 6, 7, 8 were recorded in NRe and are presented in rows for two columns of six time-binned samples for cumulative (on the left) and independent (on the right) time intervals. Cell 3 was recorded in the AM of the thalamus. All cell numbers for perimeter/border cells on this figure correspond with cells presented on Figure 4.

Units were found in NRe (and two in AM) that fired when the rat was close to a physically-defined boundary in the circular arena, potentially signaling the presence of a perimeter/border. These units met the criteria stated in the methods for us to determine the presence of a perimeter/border-related firing. Figure 4 provides examples of the firing properties of these cells; examples of single-unit activity are provided in Figure 8). These reuniens cells signal the presence of either part of the perimeter/border (Figure 4A, cells 1, 2, 4, 8, with the exception that cell 3 was recorded in the ventral part of AM) or the entirety of the perimeter/border (Figure 4A, cells 5–7). Unlike place cells, the activity of perimeter/border cells was not entrained by 6–12 Hz oscillations as depicted on ±1000 ms autocorrelograms (Figure 4A). All perimeter/border units show location-specific firing that remains stable and constant across the duration of the recording session. Figure 5 depicts the temporal evolution of firing of perimeter/border units for cumulative samples (left-hand columns) and for independent time-binned samples (right-hand columns). Perimeter/border-related activity is present from the first minute of exposure to the environment, indicating that there is a rapid or near-instantaneous expression of boundaries of the recording environment by these thalamic cells. The units from NRe (*n* = 11) had an average firing rate of 1.1 ± 0.4 Hz and a maximal firing rate of 5.7 ± 1.5 Hz, with a mean spike amplitude of 133.6 ± 1.5 μV and an average spike width of 126.3 ± 2.5 μs. We analyzed and identified these cells using the metric described above. Among 11 visually identified perimeter/border cells, 10 of them survived the criteria described above. The firing field of these cells have an angular extent of 248° ± 105.74° (min: 115°, max: 360°) degrees, with the percentage of spiking activity being 90.5 ± 5.37 (min: 82.48, max: 98.47) in proportion to the distance from the perimeter/border to the center of 0.41 ± 0.07 (min: 0.29, max: 0.54).

### Head Direction Cells are Present in the Anteromedial and Reuniens Nuclei of the Thalamus

**Figure 6:**
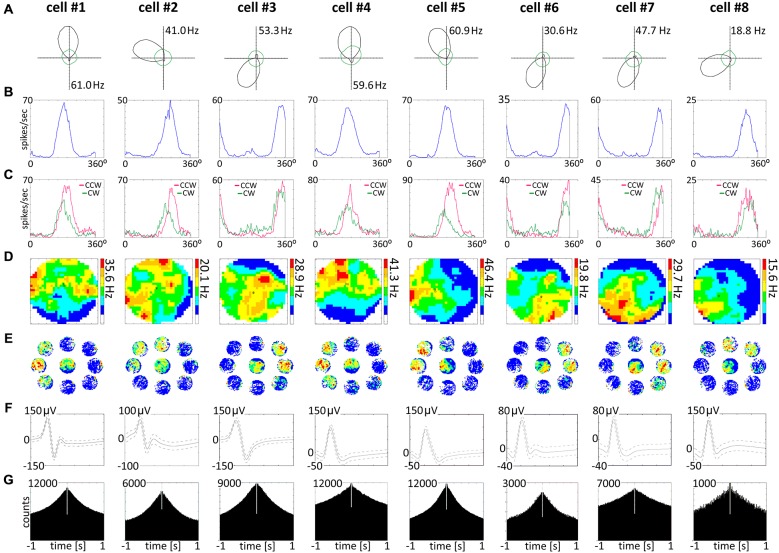
**Head direction cells recorded in the AM of the thalamus. (A)** Head direction cells with consecutive numbers are presented in columns 1–8. For each unit, data recorded during the whole 20-min sessions are presented. **(A)** Polar plots. **(B)** Tuning curves. **(C)** Tuning curves for clockwise and counter-clockwise movement of the rat, showing the separation angle observed in examples of recorded head direction cells. **(D)** Firing intensity maps. **(E)** Spatial analysis conducted for cardinal orientations showing no effect of spatial position on unit activity. **(F)** Spike waveforms of head direction cells recorded in the AM of the thalamus. **(G)** Autocorrelation ±1000 ms depicting characteristic temporal firing pattern.

Head direction cells were found in the AM thalamic nucleus (*n* = 36). A separate population of head direction cells found in NRe was described elsewhere (Jankowski et al., 2014). A sample of head direction cells recorded in the AM is presented in polar plot form (see Figure 6A, cells 1–8). These cells show firing rates in the 18–61 Hz range (similar to the firing rate of head direction cells found in other parts of the head direction system), with a characteristic triangular tuning curve representing spiking activity when firing rates are plotted on the ordinate and the animal’s head direction is plotted on the abscissa. The normalized average tuning curve of the units recorded in the AM showed well expressed head directionality (Figure 6B, cells 1–8). The AM units (*n* = 36) had an average firing rate (mean ± SEM) of 4.9 ± 1.0 Hz, with a maximal place map firing rate of 11.1 ± 2.3 Hz, a mean spike amplitude of 101.8 ± 3.3 μV, and an average spike width of 116.0 ± 1.7 μs. Average peak rate for HD cells was 20.1 ± 4.3 Hz. All recorded units demonstrated a separation angle when firing rates were separated into clockwise and counter-clockwise components (Figure 6C). There were no significant differences between mean head direction measured in degrees for clockwise and counter-clockwise movement in the *t*-test for paired two samples for means in the whole population of HD cells recorded in AM. Finally, a spatial analysis conducted for cardinal directions showed no effect of spatial position on unit activity (Figures 6D,E). Activity of these HD cells was not entrained by 6–12 Hz oscillations, as presented on ±1000 ms autocorrelations of spiking activity (Figure 6G). Examples of single-unit activity are provided in Figure 8.

### Head Direction Cells fire from First Exposure to an Environment

**Figure 7:**
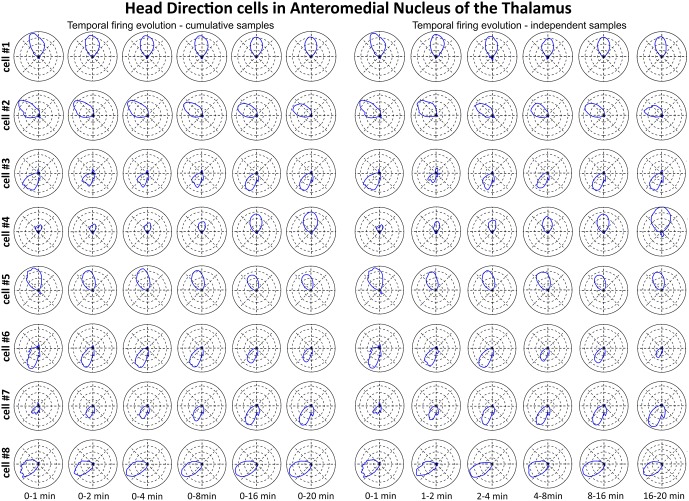
**The temporal evolution of discharge profile of head direction cells recorded in AM of the thalamus**. The temporal evolution of head-direction-related firing activity of head direction cells recorded in the AM of the thalamus was depicted in polar plots created for chosen time intervals. Cells 1–8 are presented in time-binned samples for cumulative (on the left) and independent (on the right) time intervals. All cell numbers for head direction on this figure correspond with cells presented on Figure 6.

We tested time of onset of first firing for our head direction cell samples. Figure 7 depicts the temporal evolution of head directional cells from the AM in polar plots. Head-direction-related discharge is present from the first minute of exposure to the environment, indicating that there is a rapid or near-instantaneous expression of head direction information by these thalamic cells. The expression of the directional receptive field as indexed by the polar plots shows a similar instantaneity and consistency of expression from first exposure to the environment through the extent of the recording session.

### Place, Head-Direction and Perimeter/Border Cells in the Rostral Thalamus Show within Session Stability

A particular concern for these cells is whether or not they show the locational, head-directional or perimeter/border-related activity through time, i.e., that they display the necessary degree of inter-temporal stability that characterises these spatial signals in other brain regions. Although these experiments do not address the issue of between-session stability of these spatial signals, our experiments do address the issue of stability of spatial signals within the recording sessions. We segment the evolution of spatial firing into independent time bins (from 0–1, 1–2, 2–4, 4–8, 8–16 and 16–20 min; see Figures 3, 5, 7). Spatial activity is consistently present within each recording session, across each of the independent time bins, and the spatially-related discharge is consistent in location for rostral thalamic place cells, in direction for rostral thalamus head direction cells, and adjacent the wall for perimeter/border cells.

### Place, Head-Direction and Perimeter/Border Cells in the Rostral Thalamus Show Rapid Onset and Offset of Neuronal Responding

**Figure 8:**
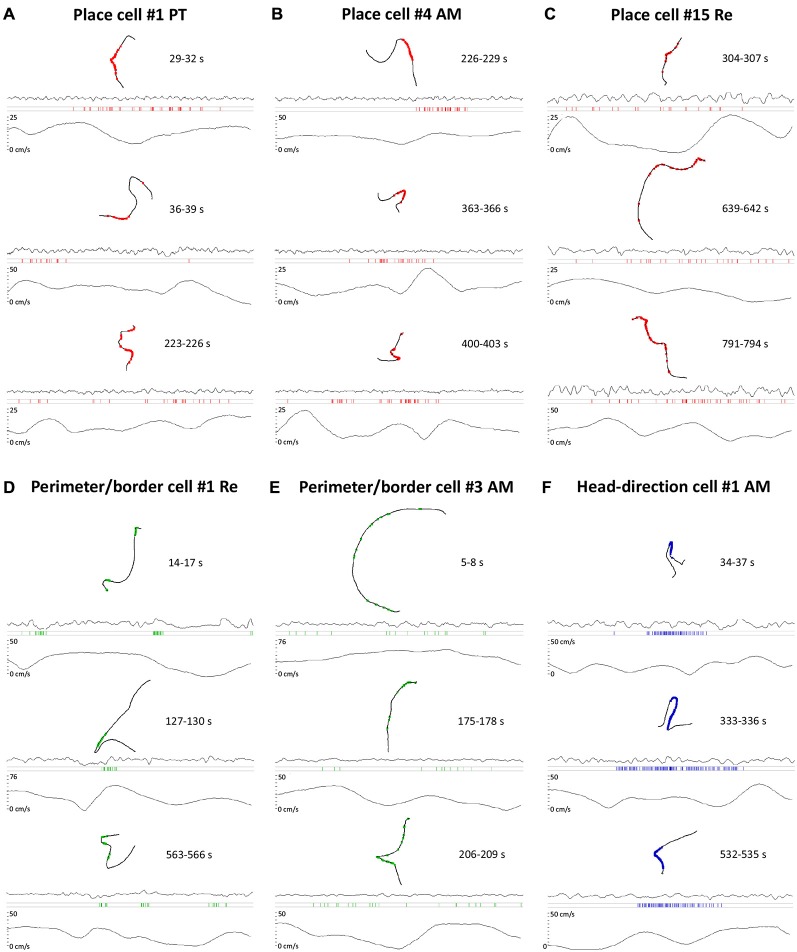
**Raw data of single units activity time locked with LFP and running speed of the rat. (A–F)** For each cell type, three representative samples of single unit activity are presented. Each sample consists of 3 s recording when the cell was active. For each sample, the following time-locked plots are presented: path of the animal with superimposed single unit activity, LFP trace, raster plot of single unit activity and running speed of the animal. On the right to the path of the animal on each plot the time of the sample extracted from 1200 s recording is shown. The activity of place cells is in red, perimeter/border cells in green and head-direction cell in blue. **(A)** Activity of place cell recorded in the PT of the thalamus when the rat was walking/running through the place field. **(B)** Activity of a place cell recorded in AM of the thalamus when the rat was walking/running through the place field. **(C)** Activity of a place cell recorded in NRe of the thalamus when the rat was walking/running through the place field. **(D)** Activity of a perimeter/border cell recorded in NRe of the thalamus when the rat was walking/running near the boundary of the experimental arena. **(E)** Activity of a perimeter/border cell recorded in the AM of the thalamus when the rat was walking/running near the boundary of experimental arena. **(F)** Activity of a head-direction cell recorded in the AM of the thalamus when rat was walking/running with head directed in the cell’s prefered firing direction in the horizontal plane. All place cells on this figure correspond with cell numbers presented on Figure 2. All cell numbers for perimeter/border cells on this figure correspond with cells presented on Figure 4. Head direction cell number on this figure correspond with cell presented on Figure 6.

A notable feature of the units described here is their rapid onset and offset of firing, irrespective of their phenotype. Figure 8 presents a sequence of simultaneous montages of path segments with overlaid hatches representing the occurence of spikes, the corresponding local-field potential, rasters of spike occurence against time, and a trace of the speed of movement of the animal for the same time period. The path segments presented are all standardised at 3 s in length. The spike onsets and offsets occur with remarkable rapidity along the relevant portion of their trajectory, with little or no firing happening before the relevant portion of the receptive field occurs for each cell type. Notably, on visual inspection, the firing activity for each unit class differs, with the highest density of activity present for the head direction units, and the lowest for the perimeter/border cells, and place cells falling into an intermediate position.

## Discussion

While sites within the rostral medial thalamus are vital for human episodic memory (von Cramon et al., 1985; Clarke et al., 1994; Van der Werf et al., 2003; Mitchell et al., 2014), it has proved difficult to identify the most critical nuclei and their respective contributions. Although neuropathological evidence has often implicated the anterior thalamic nuclei (Aggleton and Sahgal, 1993; Harding et al., 2000; Gold and Squire, 2006; Tsivilis et al., 2008; Carlesimo et al., 2011), this clinical evidence is largely indirect, relating to white matter damage or to pathologies encompassing adjacent nuclei. More targeted evidence from animal lesion experiments (including cross-disconnection studies) has confirmed a critical role for the anterior thalamic nuclei in spatial learning and memory, while also showing that these functions are hippocampal-dependent (Parker and Gaffan, 1997; Warburton et al., 2001; Henry et al., 2004). Of the rodent anterior thalamic nuclei, the electrophysiological properties of the AD nucleus, and to a lesser extent the anteroventral nucleus, are relatively well described as both contain head direction cells (Taube, 2007; Tsanov et al., 2011a, b). Additional descriptions of units within the anteroventral nucleus have emphasised the presence of theta firing cells (e.g., Vertes et al., 2001, 2004; Albo et al., 2003). In contrast, electrophysiological information concerning the AM, along with the adjacent PT and NRe, remains exceptionally scarce even though such information may help explain the potential significance of these thalamic relays for memory.

The present study recorded units in three rostral thalamic nuclei (parataenial, anteromedial, and reuniens) in freely-moving rats. Some units possessed previously unsuspected spatial properties. The various cell types (Figures 2, 4, 6) show clear similarities to the place cells, head direction cells, and perimeter/border cells (see, for example, Lever et al., 2009; Savelli et al., 2008; Solstad et al., 2008) described in hippocampal and parahippocampal regions. The thalamic place cells recorded here are visually and phenotypically similar to those found in the hippocampus, as they are punctate in appearance and fire in a relatively restricted area of the environment. The head direction cells also resemble those found in other regions, with relatively restricted receptive fields. One notable difference is their maximal firing rates tend to be lower than those of head direction cells in other brain regions (Taube, 2007). We also describe cells with a visually-apparent firing profile parallel to the physical border (or perimeter) with a firing field of least a continuous arc of 90° degrees or greater. These cells fire in a narrow band along the perimeter, and we have therefore called these cells “perimeter/border cells”. A method for quantifying such cells is also provided.

It had been predicted that the anterior thalamic nuclei process information with high spatial and temporal resolution (Aggleton et al., 2010), while information in the midline nuclei is more likely to reflect rather diffuse roles in attention (Van der Werf et al., 2002; Vertes et al., 2007; Cassel et al., 2013). NRe, for example, is often thought to be important for arousal and awareness (Van der Werf et al., 2002; Vertes et al., 2007), consistent with lesion studies that have typically failed to find specific spatial deficits but rather suggest roles in procedural learning or arousal/affective processes that prolong memories (Dolleman-van der Weel et al., 2009; Loureiro et al., 2012; Cassel et al., 2013; but see Davoodi et al., 2009). Our current findings support the first prediction (high resolution in anterior thalamic nuclei) but directly challenge the second prediction (low resolution in NRe). NRe is a principal source of excitatory thalamic inputs to the hippocampus (Vertes et al., 2007) and forms a disynaptic link between frontal cortices and the hippocampus (McKenna and Vertes, 2004; Prasad and Chudasama, 2013). While recent functional studies seemingly emphasise its role as a frontal relay (Hembrook and Mair, 2011; Xu and Südhof, 2013), the present data point to information types potentially reflecting its hippocampal inputs.

There have been a few previous hints of spatial signals from recording studies in the rostral/anterior thalamus. While exploring a wide variety of basal forebrain sites in rats placed in a small, enclosed test apparatus with a running wheel and water bottle, Mink et al. (1983) reported one neuron in dorsolateral AM that had “orienting and locomotor” correlates, as well as one other neuron in parataenial with “orienting” correlates. More recently, Welday et al. (2011) reported theta cells in more dorsally-located anterior thalamic recordings, as did Tsanov et al. (2011b). We have also previously described the presence of a population of head direction cells in NRe of the thalamus in the freely-moving rat (Jankowski et al., 2014). This population of head direction cells provides an important additional subcortical head direction signal that could potentially modulate the hippocampal CA fields directly and thus, inform spatial processing and memory. The cells in NRe maintain head directionality during light–dark transitions and in environments of different shape. These cells also establish directionality rapidly upon first entering an environment and show remarkable constancy of heading direction across days of recording. Finally, Mizumori and Williams (1993) and Enkhjargal et al. (2014) reported some AD and laterodorsal thalamic neurons that combine heading and movement direction information, along with the trajectory route during whole-body translocation, again suggesting the presence of a possible spatial signal in the thalamus.

### How Does Place Information Reach the Rostral Thalamus?

There are at least three possible explanations. The first is that the hippocampal formation provides information necessary for the spatial activity observed in the rostral thalamus or that the rostral thalamus provides spatial information that may be necessary for the spatial activity observed in the hippocampal formation. The second possibility is that hippocampal and rostral thalamic spatial systems operate in parallel (cf. the accessory optic system). The third possibility is that there is a reciprocal inter-dependent relationship between these spatial nodes. Consistent with the first explanation, perimeter/border cells, which signal the presence of a physical border in or perimeter of an environment, are found within the subicular cortices (Lever et al., 2009) and hence, in a region projecting directly to the rostral thalamus. For place cells, the situation appears more complex. Hippocampal place cells fire in relation to the animal’s location in space (O’Keefe and Dostrovsky, 1971) and have been a key discovery in our understanding of the brain systems involved in spatial information processing. Nevertheless, there is an apparent lack of place cells in other sites receiving direct hippocampal inputs, such as prefrontal cortex (e.g., Gemmell et al., 2002). Within the hippocampal formation, place cells are mainly confined within the hippocampus proper. While some subicular units (Anderson and O’Mara, 2003) are categorised as “place cells” (Sharp, 1997, 2006; Brotons-Mas et al., 2010), they often have receptive fields larger than those found in CA1 (O’Mara et al., 2001; O’Mara, 2005). The rostral thalamic spatial cells we report here have field sizes more akin to dorsal CA1 cells, yet the overwhelming majority of direct hippocampal projections to the rostral thalamus arise from the subicular cortices (Swanson and Cowan, 1977; Wright et al., 2013). While there are very light, scattered projections from CA1 that reach NRe, the interanteromedial nucleus, the anteromedial, paraventricular, and parataenial nuclei, these inputs arise from the ventral hippocampus (Cenquizca and Swanson, 2006). One possibility for the anteromedial thalamic nucleus is that the thalamic place information arises from the proximal subiculum, which preferentially projects to the anteromedial thalamic nucleus (Wright et al., 2013) and contains cells with spatial properties more similar to those in CA1 than found in the distal subiculum (Kim et al., 2012). The subiculum inputs to NRe arise, however, from the subiculum zone midway between the proximal and distal regions (Witter, 2006), i.e., less akin to CA1 (Kim et al., 2012). Consequently, there logically remains the possibility that the spatial information found in rostral thalamic nuclei has a very different source. Suggestive evidence comes from the remaining spatial capacities seen in decorticate rats (Whishaw, 1990), from navigating rats with total hippocampal ablations (Hollup et al., 2001a,b), from preserved spatial memory after hippocampal lesions enabled by enriched environments (Winocur et al., 2005), and from learning under errorless-learning regimes in amnestic humans (Baddeley and Wilson, 1994).

One potential concern is that the cells we describe are apparently small in proportionate or percentage terms. One hypothesis is that if we analyze the data according to some categorical criterion or set of criteria, it is possible that one might find at least some units fitting into any one category by chance alone. We do not find such an argument compelling, because it would imply that a fraction of units recorded in visual cortex, motor cortex or wherever would be place cells, perimeter/border cells, or head direction cells (or whatever). This is an empirical prediction which is unsupported in the literature. A more likely explanation is that our current experiments set a lower bound to the proportions of cells types found, and that their very reliability within and across sessions implies that they are consistently present. The seeming absence of grid cells is also compelling, as it suggests that the cells we describe here might be more elemental in nature and that grid cells are in part at least constructed from these more elemental inputs.

A currently open question concerns the activity of the units that we have recorded that do not appear to be spatially-responsive or spatially-modulated. It remains possible, consistent with previous theories, that this subpopulation of rostral thalamic cells plays roles in arousal and attention; our current experiments do not directly test this possibility. Qualitatively, we did not observe any change in neuronal discharge when food pellets were thrown into the environment, despite the animal orienting to, and foraging for, the food pellets. Because it is possible to reliably record unit activity in these nuclei, it should be possible to directly test this hypothesis, especially during sleep-wake transitions, and during the presentation of arousing or orienting stimuli. Other possibilities exist also. One especially intriguing possibility is that these rostral thalamic spatial cells may act in concert with non-spatial rostral thalamic cells to facilitate the early integration of spatial representations with arousal or emotional function. These cells may also play roles in long-range oscillatory coupling, homeostatic function and co-ordination between cortical and subcortical regions, or indeed a variety of other possibilities that remain to be described. It remains possible that these nuclei subserve a plurality of functions, from the proto-cognitive ones we describe here, to a wide variety of other roles, given their central location and substantial connectivity with other brain regions.

### Conclusion

The present findings demonstrate the existence of a hitherto unsuspected population of spatial cells in the rostral thalamus, which display many of the properties of hippocampal and retro-hippocampal place cells, head direction cells and perimeter/border cells. While the precise functional role of these spatial cells within the brain’s navigational system(s) remains to be determined, the results complement those of lesion studies, which have repeatedly demonstrated that rostral thalamic nuclei are vital for normal spatial learning and memory.

## Author Contributions

SMO and JPA conceived and directed these experiments; MMJ and JP conducted experiments and analyzed data; MNI wrote Matlab scripts, derived equations and analyzed data; SMO, JPA, SLV, JE, MNI and MMJ co-wrote the paper.

## Conflict of Interest Statement

The authors declare that the research was conducted in the absence of any commercial or financial relationships that could be construed as a potential conflict of interest.
